# Europäische Währungsunion: schlecht gerüstet für große Krisen

**DOI:** 10.1007/s10273-021-2921-6

**Published:** 2021-05-17

**Authors:** Michael Heine, Hansjörg Herr

**Affiliations:** FB Wirtschaftswissenschaften, Hochschule für Wirtschaft und Recht Berlin, Badensche Straße 50-51, 10825 Berlin, Deutschland

**Keywords:** E6, H12, J3

## Abstract

Bereits vor der Corona-Krise zeigte die Europäische Währungsunion (EWU) eine unbefriedigende wirtschaftliche Entwicklung mit niedrigem Wachstum und zu geringer Inflation. Zur Krisenbekämpfung ist die Koordination zwischen Geld- und Fiskalpolitik notwendig. Unzureichende Lohnsteigerungen, die zu Deflation führen, müssen vermieden werden. Nicht zuletzt ist ein Mechanismus zur Stabilisierung des Finanzsystems und zum schnellen Umgang mit notleidenden Krediten erforderlich. Abgesehen von der Geldpolitik fehlt es der EWU an Institutionen, welche die notwendige Wirtschaftspolitik unterstützen. Es besteht somit die Gefahr einer langfristigen Stagnation in der EWU.

## Abstract (en)

Even before the COVID-19 crisis, the European Monetary Union (EMU) exhibited an unsatisfactory economic development with low growth and inflation rates below the target rate of the central bank. Macroeconomic coordination among monetary and fiscal policy is necessary to fend off such large crises. Insufficient wage increases that lead to deflation need to be avoided. Last, but not least, a mechanism is needed to stabilise the financial system and quickly deal with non-performing loans. Except in the field of monetary policy, the EMU lacks institutions that support quick and sufficient economic policies. There is the danger that policy mistakes will lead to long-term stagnation in the EMU or possibly even a great recession.

Wenngleich es bereits in der zweiten Hälfte 2019 zu einem konjunkturellen Abschwung in der Europäischen Währungsunion (EWU) gekommen war, hat die Corona-Pandemie diese Abwärtsbewegung erheblich beschleunigt. Das Bruttoinlandsprodukt (BIP) ist seit der Großen Depression in den 1930er Jahren in Europa nicht mehr so stark gesunken wie 2020. Bekanntlich sinken bei wirtschaftlichen Abschwüngen die staatlichen Einnahmen, und es steigen die Ausgaben. Daher ließ sich die Schuldenbremse, ohne desaströse Konsequenzen zu riskieren, nicht verteidigen. Sie wurde außer Kraft gesetzt. Damit hat die Fiskalpolitik erstmals seit der Finanzmarktkrise 2007/2008 wieder an einem Strang gezogen mit der seit Jahren expansiv ausgerichteten Geldpolitik.

Obwohl die wirtschaftlichen Folgen der Pandemie noch nicht abzuschätzen sind, ist vor allem in Deutschland eine heftige Diskussion entbrannt, wie es mit der Schuldenregel weitergehen soll. Z.B. hat der eher moderate Vorschlag von Kanzleramtschef Helge Braun, die Schuldenbremse zeitweilig auszusetzen und dazu das Grundgesetz zu ändern, heftige Kritik ausgelöst. Mit sehr unterschiedlichen Begründungen wurde stattdessen etwa vom haushaltspolitischen Sprecher der CDU-Fraktion im Bundestag, Eckhardt Rehberg, oder auch vom ifo-Präsidenten Clemens Fuest gefordert, möglichst schnell zur Schuldenbremse und Haushaltskonsolidierung zurückzukehren (Tagesschau, 2021). Sollte Deutschland – wahrscheinlich gemeinsam mit seinen nordeuropäischen Verbündeten – als das ökonomisch stärkste und zugleich stabiles Land bereits 2022 diesem Rat folgen, würden so die Weichen für die EWU insgesamt entsprechend gestellt.

Die aktuelle Diskussion konzentriert sich auf die Ausgestaltung der Fiskalpolitik. Das ist für die künftige Entwicklung der EWU ohne Zweifel ein wichtiger Bereich. Aber diese Diskussion greift zu kurz, da so zentrale Problemfelder aus den Augen verloren werden, wie die Gefahren der hohen Verschuldung des staatlichen, jedoch vor allem privaten Sektors in einer Reihe von EWU-Ländern. Sollte die EWU in eine Stagnationsphase gleiten, dürfte dies zu einem scharfen Anstieg von notleidenden Krediten führen. Ein weiteres und völlig aus dem Blickfeld geratenes Problem ist der krisenbedingte Druck auf die Lohnentwicklung, der zu einer deflationären Entwicklung massiv beitragen kann. Dies zeigen nicht nur theoretische Erwägungen, sondern auch ein Blick in die Wirtschaftsgeschichte. Gerade die Große Depression, die nach 1929 einsetzte, aber auch die seit 20 Jahren hartnäckige Stagnation der japanischen Volkswirtschaft belegen eindrucksvoll, wie gefährlich deflationäre Entwicklungen sind.

**Prof. em. Dr. Michael Heine** lehrte Volkswirtschaftslehre und war Präsident an der Hochschule für Technik und Wirtschaft Berlin.

**Prof. em. Dr. Hansjörg Herr** lehrte Volkswirtschaftslehre an der Hochschule für Wirtschaft und Recht Berlin.

## Deflationsgefahren bei geringem Wachstum

Die wirtschaftliche Entwicklung in der EWU verläuft bereits seit der Finanzmarktkrise 2007/2008 unbefriedigend. Das durchschnittliche jährliche Wachstum des realen BIP betrug in der EWU von 2008, dem ersten Jahr der Großen Rezession, bis 2019 nur gut 1 % (vgl. Abbildung 1). Die Unterschiede zwischen den Mitgliedsländern waren erheblich. Während die EWU von 2007 bis 2019 insgesamt um 9,5 % wuchs, lagen die Werte z. B. für Deutschland bei 14,6 %, für Frankreich bei 11,4 %, für Spanien bei 6,6 %, für Portugal bei 4,9 %, für Italien bei -0,03 % und für Griechenland bei -23,2 % (OECD, 2021). Nach Zahlen der Europäischen Zentralbank (ECB, 2020) dürfte das BIP 2020 in der EWU insgesamt um 7,3 % geschrumpft sein. Auch hier verzeichnet Deutschland mit -5,5 % im Vergleich zu Portugal mit -8,1 %, Italien mit -9 %, Frankreich mit -9,3 %, Griechenland mit -10 % und Spanien mit -11,1 % relativ gute Werte. Für 2021 erwartet die EZB eine Erholung des BIP von 3,9 %.

Abbildung 1

**Vierteljährliche Veränderungen des realen BIP, der Inflationsrate und der Nominallöhne in der Eurozone**

jeweils 1. Quartal

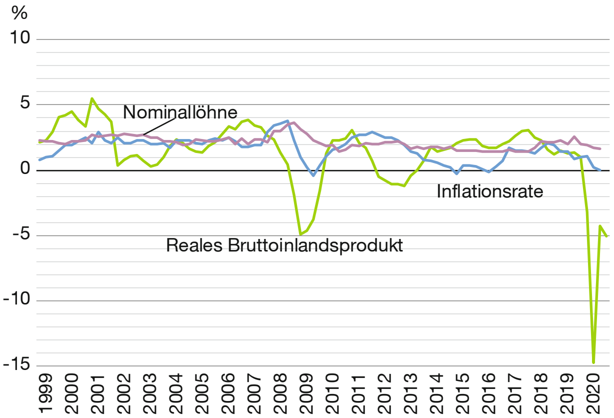

Quellen: ECB (2021); Eurostat (2021).

Vor dem Hintergrund dieser Entwicklung überrascht es nicht, dass die Arbeitslosigkeit in der EWU hoch ist und 2020 auf 8,3 % anstieg. Dabei lagen – wie zu erwarten – Länder wie Griechenland mit 18 %, Spanien mit 16,7 %, Italien mit 9,9 % oder Frankreich mit 8,5 % deutlich vor Deutschland (4 %) (EK, 2020). Die schleppende Konjunktur spiegelte sich auch in den Veränderungen des Preisniveaus wider. 2003 hatte die EZB ihr damaliges Inflationsziel, das zwischen Null und 2 % lag, aufgrund sehr niedriger Inflationsraten vor allem in Deutschland (2003 rund 1 %) und wegen der Furcht, man könne bei einer Inflationsrate von nahe Null schnell in eine Deflation abrutschen, auf knapp unter 2 % korrigiert. Damit sollten deflationäre Gefahren vermieden werden (Heine und Herr, 2021). Dieses Inflationsziel hat die EZB ab 2013 trendmäßig deutlich verfehlt (vgl. Abbildung 1). 2020 betrug die Inflationsrate in der Eurozone 0,2 %, und einige Euroländer waren mit einem fallenden Preisniveau konfrontiert. In Griechenland fiel es um 1,3 %, in Zypern um 1,2 %, in Estland und Irland um 0,6 % und in Italien, Portugal und Slowenien um 0,2 %. Für 2021 erwartet die EZB für die Währungsunion erneut eine sehr niedrige Inflationsrate von 1 % und für 2022 von 1,1 % (ECB, 2020).

Der Hauptgrund für die niedrige Inflationsrate liegt in den zu geringen Lohnerhöhungen. Aufgrund der unbefriedigenden ökonomischen Entwicklung nach der Großen Rezession blieb der Druck auf die Tarifparteien hoch, gemäßigte Lohnabschlüsse zu vereinbaren. Von 2011 bis 2020 sind die Nominallöhne je Beschäftigten in der EWU jährlich nur um 1,7 % gestiegen. Aber auch hier gab es Unterschiede zwischen den Ländern. So stiegen sie in Deutschland um 2,3 %, in Italien um 0,9 % und in Spanien um 0,7 %. In Griechenland sanken sie in diesem Zeitraum im Jahresdurchschnitt um 2,3 % (EK, 2020). Die Entwicklung der Nominallöhne spiegelt sich in der Entwicklung der nominalen Lohnstückkosten wider. Denn Lohnerhöhungen lassen sie steigen und Produktivitätsfortschritte lassen sie sinken. Die Entwicklung der Lohnstückkosten ist nach Keynes (1930) der wichtigste Faktor für die Preisniveauentwicklung. Die nominellen Lohnstückkosten erhöhten sich in der EWU von 2011 bis 2019 jährlich durchschnittlich nur um 1,09 %. In Deutschland stiegen sie überdurchschnittlich an, nachdem sie von Mitte der 1990er Jahre bis zur Großen Rezession stagnierten. Geringe Erhöhungen verzeichneten beispielsweise Frankreich und Italien. In Spanien war der Anstieg der Lohnstückkosten null, und Griechenland musste fallende Lohnstückkosten hinnehmen (Eurostat, 2021). Aufgrund dieser Entwicklung konnte die EZB ihr Inflationsziel nicht realisieren.

Funktional wäre eine Erhöhung der Nominallöhne gewesen, die der gestiegenen Trendproduktivität plus der Zielinflationsrate der EZB entspricht. Die Arbeitsproduktivität stieg in der EWU von 2011 bis 2019 jährlich um durchschnittlich 0,81 % (OECD, 2021). Somit hätten die Nominallöhne bei einer Zielinflationsrate von etwa 1,9 % um durchschnittlich rund 2,7 % und nicht nur um 1,09 % steigen müssen. Bei einer solchen Abweichung steht eine Zentralbank auf verlorenem Posten. Am Rande sei erwähnt, dass es noch weitere Deflationstreiber gab. Der Preis für Rohöl stürzte von einem Niveau über 100 US-$ pro Barrel Ende 2014 auf ein Niveau von derzeit unter 60 US-$ ab, was die niedrige Inflationsrate weiter begünstigte (OECD, 2021).

Es besteht die Gefahr, dass in den nächsten Jahren, insbesondere bei einer stagnierenden Entwicklung, der nominelle „Lohnanker“ bricht. Denn die mikroökonomische Logik, wonach Unternehmen durch Lohnzurückhaltung gerettet werden müssen, erscheint in der Krise überaus plausibel. Sie dürfte in besonders betroffenen Krisenregionen noch an Überzeugungskraft gewinnen, da die Versuchung naheliegt, die Krise durch eine interne Abwertung mittels sinkender Löhne zu meistern. Im Ergebnis würde es freilich nicht zur Überwindung der Krise, sondern zur Deflation kommen. Sie wiederum erhöht die Realschulden der Unternehmen, führt zur Explosion fauler Kredite und lähmt die Investitionsneigung der Unternehmen. Eine gefährliche Negativspirale kann in diesem Fall nicht ausgeschlossen werden.

## Geldpolitik

An dem geringen Wachstum und dem Verfehlen des Inflationsziels konnte auch die expansive Geldpolitik der EZB nichts ändern (Heine und Herr, 2021). Der Hauptrefinanzierungszinssatz wurde von der EZB ab 2012 schrittweise gesenkt. Im März 2016 erreichte er den Wert von Null und verblieb bis heute auf diesem Niveau. Die expansive Zinspolitik wurde mit einer „unkonventionellen Geldpolitik“ verbunden, deren Darstellung den Rahmen dieses Beitrags sprengen würde (Heine und Herr, 2021). Das Ausmaß der verschiedenen Maßnahmen ist jedenfalls gewaltig. Die Bilanzsumme der EZB betrug zwischen 1999 und 2005 um die 10 % am BIP der EWU und stieg dann bis 2009 auf etwa 15 %. Bis 2019 erreichte sie fast 40 % und Ende 2020 belief sie sich auf rund 50 % (ECB, 2021a). In diesem Rahmen hat die EZB die öffentlichen Haushalte indirekt in großen Umfang refinanziert und dadurch die Zinsen auf Staatspapiere sehr niedrig gehalten – in Deutschland realisieren zehnjährige Staatsanleihen ab 2019 einen negativen Zinssatz (OECD, 2021). Anfang 2021 hielt die EZB 25 % der gesamten öffentlichen Schulden in der Eurozone.

Die „Easy Money Policy“ der EZB konnte insbesondere das Investitionsverhalten der Unternehmen nicht nachhaltig verändern. Die realen Bruttoanlageinvestitionen stiegen von 2011 bis 2020 in der EWU jährlich um durchschnittlich 0,7 %. In Deutschland betrug dieser Wert auch nur knapp 2 %, in Italien -2,1 %, in Spanien -1,2 % und in Griechenland sogar -7,1 % (EK, 2020). Es zeigt sich an diesen Entwicklungen die asymmetrische Macht einer Zentralbank: Zwar vermag sie durch eine zunehmend restriktive Geldpolitik private Investitionen zurückzudrängen, aber sie kann im Rahmen eines rezessiven Umfelds beim Versuch, positive Erwartungshaltungen bei den Wirtschaftssubjekten zu generieren, scheitern.

Die EZB, die ab 2012 insgesamt geldpolitisch richtig gehandelt hat, konnte allerdings ohne die Unterstützung der Fiskal- und Lohnpolitik Kollateralschäden nicht vermeiden. So hat sie die Gefahren von Blasen auf den Aktien- und Immobilienmärkten deutlich erhöht (vgl. Abbildung 2). Die Entwicklung der Aktienkurse in der EWU ist beunruhigend. Anfang 2021 wurde ein Kursniveau erreicht, das mit dem Höhepunkt der Internetblase Ende der 1990er Jahre und der Spekulation vor der Finanzmarktkrise 2008 vergleichbar ist. Auch die Entwicklung der Immobilienpreise deutet auf eine starke Vermögensmarktinflation hin. Zwar sind nach der Großen Rezession die Immobilienpreise in einer Reihe von Ländern gesunken oder stagnierten, jedoch ist ab etwa 2015, mit Ausnahme von Ländern wie Italien oder Griechenland, in den meisten Ländern ein deutlicher Anstieg der Immobilienpreise zu beobachten. Von 2015 bis zum letzten Quartal 2019 betrug der Anstieg in Deutschland 33 %, in Frankreich 12 %, in Spanien 26 %, in den Niederlanden 35 %, in der Slowakischen Republik 38 % oder in Portugal 45 % (OECD, 2021). Sollten die Blasen platzen und sich so eine Vermögensmarktdeflation mit einer Gütermarktdeflation verbinden, wäre das keine ermutigende Perspektive.

Abbildung 2
**Entwicklung der Aktien- und Immobilienmärkte in der Eurozone**
2015 = 100, jeweils 1. Quartal

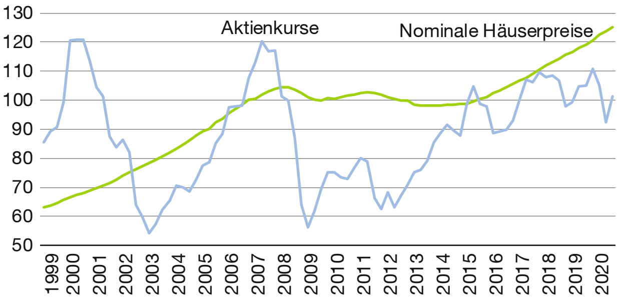

Quelle: OECD (2021).

## Fiskalpolitik

Zwingend notwendig für eine erfolgreiche Wirtschaftspolitik ist die Kooperation zwischen der Geld- und der Fiskalpolitik. Gegen diese Erkenntnis wird in der EMU regelmäßig verstoßen. Betrachten wir die Große Rezession. Die EZB schaltete zwar erst 2008, dann aber entschieden auf einen expansiven Kurs um. Zeitgleich wurden in den großen Volkswirtschaften wie in Deutschland Konjunkturpakete beschlossen und umgesetzt. Im Ergebnis konnte die Krise schnell überwunden werden. Aber bereits im Verlauf von 2009 wurden in Deutschland die Weichen neu gestellt und die Schuldenbremse ins deutsche Grundgesetz übernommen. Gleichzeitig erhöhte Deutschland erfolgreich den Druck auf andere Länder der Eurozone, ihre öffentlichen Budgetdefizite ebenfalls zu konsolidieren

(vgl. Abbildung 3). Spätestens nach dem Ausbruch der sogenannten Staatsschuldenkrise 2010 folgte die ganze EWU einem dysfunktionalen fiskalischen Sparkurs. Die Folge war eine weitere Rezession 2012 und 2013. 2012 wurde der Fiskalvertrag unterzeichnet, der alle EWU-Länder unter anderem verpflichtet, keine strukturellen Defizite von mehr als 0,5 % am BIP zuzulassen und die Verschuldungsquote der öffentlichen Haushalte auf 60 % zu reduzieren (Blyth, 2013).

Auch mit Beginn der Corona-Krise schaltete man in der EWU auf eine expansive Fiskalpolitik um und zog mit der EZB an einem Strang. Erwähnenswert ist in diesem Kontext, dass sich die Finanzminister der EWU im Frühjahr 2020 nach zähen Verhandlungen auf ein gemeinsames Finanzpaket in Höhe von 540 Mrd. Euro geeinigt haben. Ein wichtiger Schritt in Richtung einer Fiskalunion ist der Wiederaufbauplan vom Juli 2020. Die EU beschloss zum ersten Mal, in relevantem Umfang von 750 Mrd. Euro gemeinsam Kredite aufzunehmen. Davon werden 390 Mrd. Euro als Zuschüsse an Staaten gewährt, die besonders von der Krise betroffen sind. Die restlichen 360 Mrd. Euro werden als Kredite vergeben. Schließlich wurde die Schuldenbremse außer Kraft gesetzt (Heine und Herr, 2021).

Zur Beurteilung der künftigen Entwicklung muss beachtet werden, dass es sich bei den Corona-Programmen um einmalige Aktionen handelt und es daher in der Fiskalpolitik auch weiterhin keinen EWU-weiten Partner für die EZB gibt. Zudem wurde bei der mittelfristigen Finanzplanung bis 2027 keine Stärkung des fiskalischen Zentrums der EU beschlossen, obwohl es im Vergleich zu andern Währungsräumen äußerst schwach ist. Die Fiskalpolitik bleibt eigenständige Angelegenheit der Mitgliedsländer. Eine solche Ausgestaltung eines Währungsraums ist einmalig in der Welt und erschwert eine gemeinsame Fiskalpolitik.

## Schulden und faule Kredite

Es ist zurzeit noch nicht absehbar, ob die EWU erneut, wie nach der Großen Rezession, zu früh eine expansive Fiskalpolitik aufgibt und damit Wachstumschancen verspielt. Es ist nicht auszuschließen, dass vor allem Deutschland im Verbund mit den nordeuropäischen Partnern die wachsende Verschuldung der öffentlichen Haushalte in einigen Ländern der Eurozone zum Anlass nimmt, den Fiskalvertrag schnell wiederzubeleben. 2000 betrug die staatliche Verschuldung in der Eurozone 69 % des BIP. Während der Großen Rezession stieg sie an und konnte danach, trotz aller Konsolidierungsversuche, nur marginal auf 84 % 2019 reduziert werden (vgl. Abbildung 3). 2020 stieg sie dann auf über 100 %. Die Unterschiede sind jedoch beachtlich. Ende 2020 betrug die Verschuldung des öffentlichen Sektors z. B. in Deutschland 71 %,

Abbildung 3

**Verschuldung der Unternehmen, des Staates und der privaten Haushalte in der Eurozone**

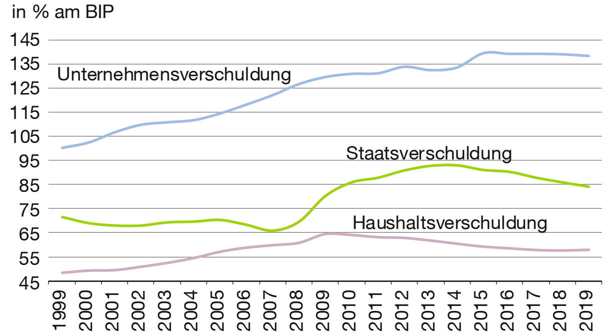

Quellen: BIS (2021); Eurostat (2021); Europäische Zentralbank (2021).

in Frankreich 116 %, in Spanien 120 %, in Portugal 135 %, in Italien 160 % und in Griechenland 207 % (EK, 2020).

Zugenommen haben in der EWU auch die Verschuldungsquoten des Unternehmenssektors (ohne Finanzinstitutionen) und der privaten Haushalte. Deren Verschuldung stieg zusammengenommen von 121,4 % am BIP 1999 auf 174 % 2020. Zu einem starken Anstieg kam es bis zur Großen Rezession. Bei den privaten Haushalten konnte sie danach leicht zurückgeführt werden, bei den Unternehmen stieg sie weiter leicht an. Sie verharrt seit 2008 auf einem relativ hohen Plateau (vgl. Abbildung 3). Wird der Staat mitberücksichtigt, erhöhte sich die Gesamtverschuldung aller drei Sektoren von 190,4 % am BIP 1999 auf 218,8 % 2007. Im zweiten Quartal 2020 lag sie schließlich bei beachtlichen 269 %. Besonders hoch waren die Gesamtverschuldungsquoten 2020 in Frankreich (347 %), den Niederlanden (316,9 %), Griechenland (301,3 %), Irland (297,7 %), Portugal (293,2 %) und in Spanien (273,5 %). In Deutschland lag der Wert bei 186,6 %. Zum Vergleich: In den USA lag er bei 270,8 % und in Japan bei 397 % (BIS, 2021).

Dieser trendmäßige Schuldenaufbau kann die künftige ökonomische Entwicklung erheblich belasten. Denn falls Unternehmen und private Haushalte ihre Verschuldung als hoch ansehen und zu reduzieren versuchen, belasten sie durch rückläufige Konsum- und Investitionskäufe die effektive Nachfrage und bremsen so auch eine expansive Geldpolitik aus. Zudem erhöhen steigende Verschuldungsquoten aus Sicht der Kreditgebenden das Risiko von Kreditausfällen. Dadurch werden Kredite zurückhaltender angeboten (ECB, 2017, 55 f.). So hat Hymen Minsky (1975) mit aller Deutlichkeit gezeigt, dass eine Phase steigender Verschuldungsquoten mit hoher Wahrscheinlichkeit in eine Vermögensmarktdeflation und ökonomische Krise münden wird.

Hohe Verschuldungsquoten erschweren auch Maßnahmen gegen inflationäre und deflationäre Entwicklungen. Bei Inflationen – wie bei Spekulationsblasen – stehen Zentralbanken vor dem Dilemma, dass steigende Zinssätze Schuldner:innen in die Knie zwingen und so eine konjunkturelle Krise eingeleitet wird. Je höher die Schuldenbestände, umso gravierender die Krise. Verbinden sich hohe Verschuldungsquoten umgekehrt mit einem sinkenden Preisniveau, so steigt die reale Schuldenbelastung. Ein Anstieg der faulen Kredite und Insolvenzen erschüttert dann das Finanzsystem und mithin die gesamte Ökonomie. Selbst ein Rückgang der Zinssätze kann in einer solchen Konstellation den Anstieg der realen Schuldenbelastung nicht kompensieren. Um es mit Irving Fisher (1933) zu sagen, schwankt dann das ökonomische Boot nicht nur, sondern es kann kentern.

Ohne Zweifel wird die Corona-Krise zu einem Anstieg fauler Kredite führen. Die EZB geht bei einem negativen, aber plausiblen Szenario davon aus, dass sich die faulen Kredite in der EWU 2022 auf 1,4 Billionen Euro (deutlich über 10 % des BIP) akkumulieren könnten. Mitte 2020 beliefen sie sich noch auf 0,55 Billionen Euro. Besonders problematisch ist, dass sich die faulen Kredite schwerpunktmäßig in den Ländern sammeln, die bereits eine hohe Staatsverschuldung aufweisen (ECB, 2021b) und folglich relativ wenig Potenzial für Gegenmaßnahmen besitzen. Vor dem Hintergrund der Erfahrungen durch die Staatsschuldenkrise besteht die Gefahr, dass sich die vergleichsweise stabilen Länder wie Deutschland oder die „sparsamen Vier“ weigern, den Krisenländern zu helfen.

Eine an sich notwendige EWU-weite Lösung steckt noch in den Kinderschuhen. Im Rahmen der Europäischen Bankenunion wurde 2015 zwar ein einheitlicher europäischer Bankenabwicklungsmechanismus beschlossen und ein europäisches Abwicklungsgremium geschaffen. Letzteres hat zur Unterstützung seiner Aufgaben einen Abwicklungsfonds zur Verfügung, der schrittweise aufgebaut und 2023 0,06 Billionen Euro oder 1 % der Bankeinlagen umfassen soll (Single Resolution Board, 2020). Dass dieser Betrag bei einer ernsthaften Krise nicht ausreichen wird, liegt auf der Hand. Da es zudem keine gemeinsame Einlagenversicherung in der EWU gibt, dürfte es dann auch wieder zu massiven Verlagerungen von Einlagen aus Krisenländern in stabilere Länder kommen. Ungemach droht der EWU auch auf der globalen Ebene. Im neuesten Report der Weltbank (World Bank, 2021), der 69 Länder umfasst, werden die erheblichen Risiken, die aus der externen Verschuldung von Entwicklungsländern resultieren, deutlich herausgearbeitet. Kommt es hier zur Krise, bleibt die EWU gewiss nicht ungeschoren. Zusammenfassend sieht die Lage der Eurozone alles andere als rosig aus. Es existieren unzweifelhaft erhebliche Risiken, und es kann nicht ausgeschlossen werden, dass die ökonomische Lage außer Kontrolle gerät.

## Lehren aus der Großen Depression

Selbstverständlich befinden sich die Mitgliedstaaten der EWU in einer anderen ökonomischen Situation als vor der Großen Depression. So gibt es keinen Goldstandard mehr, der die Geldpolitik einer Reihe von Ländern einengte. Es gibt auch keine destabilisierende Verschuldung zwischen den entwickelten Ländern insbesondere aufgrund der Reparationsverbindlichkeiten Deutschlands als Resultat des Versailler Vertrags von 1919. Gleichwohl finden sich einige interessante Parallelen. Nehmen wir die USA in den 1920er Jahren. Aufgrund ihrer international dominanten Stellung besaß sie große geldpolitische und fiskalische Spielräume. In den 1920er Jahren entwickelte sich in den USA eine Spekulationsblase auf den Aktienmärkten und auch die Immobilienmärkte zeigten Übertreibungen. Beispielsweise hatte sich der Wert des Dow Jones Industrial Average Index von 1925 bis 1929 verdreifacht. Die Verschuldungsquoten von Unternehmen und privaten Haushalten stiegen stark an. Obwohl kein Zweifel bestand, dass es sich um eine Blase handelte, war es innerhalb der US-Zentralbank (Fed) höchst umstritten, mit welchen Maßnahmen man der Blase Luft entziehen sollte. Während die eine Fraktion Zinserhöhungen vorschlug, wollte die andere die Kreditvergabe für Spekulationszwecke beschränken (Ahamed, 2018). Letztlich blieben die Zinssätze niedrig und man tat nichts. Für diese Linie sprach scheinbar, dass die Inflationsrate Ende der 1920er Jahre sehr niedrig wurde und auch die Rohstoffpreise deflationäre Entwicklungen zeigten.

Als die Aktienblase 1929 platzte und eine Vermögensmarktdeflation einsetzte, kam es zur Krise. Im Kampf dagegen wurden allerdings massive Fehler begangen, sodass es zur Großen Depression und einer rund zehnjährigen Stagnationsphase kam. Das reale BIP von Ende 1929 konnte erst 1940 in den USA wieder erreicht werden (Ahamed, 2018; Dodig und Herr, 2015). Welche Fehler waren es, die aus einer zyklischen Krise eine Große Depression machten?

1. Auf die Krise wurde fiskalpolitisch nicht reagiert. Im Gegenteil wurde zunächst versucht, die öffentlichen Haushalte zu konsolidieren. Im Ergebnis blieb der staatliche Budgetsaldo bis 1932 in etwa ausgeglichen und wurde erst mit dem Beginn der Roosevelt-Regierung im März 1933 negativ. Allerdings blieben die Budgetdefizite moderat und pendelten zwischen maximal 5,4 % (1934) und 0,1 % (1938) in Relation zum BIP. Erst 1942, mit dem Einstieg in den Zweiten Weltkrieg, stieg das Budgetdefizit auf 12,7 % und leitete eine langjährige Wachstumsphase ein.

2. Die Geldpolitik reagierte inadäquat. Als die Fed ab 1929 doch versuchte, der Spekulation mit steigenden Zinssätzen entgegenzuwirken, war es zu spät. Im Oktober 1929 platzte die Blase, wodurch die Banken aufgrund der Kreditausfälle in Schwierigkeiten gerieten. Die Fed reagierte auf diese Herausforderung in der Tradition von Bagehot (1873), wonach Banken bei Liquiditätsengpässen, nicht aber bei Solvenzkrisen, geholfen werden sollte. Selbst für eine Zentralbank ist es in der Praxis schwer, zwischen einer Liquiditäts- und einer Solvenzkrise zu unterscheiden. Für Bankkund:innen ist eine solche Unterscheidung unmöglich. Folgerichtig kam es zu Bank Runs. Im Ergebnis brachen zwischen 1930 und 1933 ca. 9.000 Banken zusammen.

3. Es kam zu einem deflationären Prozess. Die Nominallöhne sanken in den USA nach 1929 um mehr als 20 % und trieben den Deflationsprozess an. Dies verschärfte die Finanzmarktkrise entscheidend, da es so zahlreichen Schuldner:innen unmöglich wurde, ihren Verpflichtungen nachzukommen.

4. Viele Länder kamen ins Trudeln, da sie in den USA hoch verschuldet waren. Die US-Kapitalexporte drehten sich 1928 und machten meist hohen spekulativen Kapitalimporten in die USA Platz. Die US-Regierung und die Fed versäumten es, die betroffenen Staaten mit langfristigen Krediten, kurzfristigen Liquiditätshilfen und Exportmöglichkeiten zu stabilisieren. Stattdessen erhöhten die USA mit dem Smoot-Hawley-Zollgesetz 1930 ihre Zölle kräftig. Die USA wollten die Weltwirtschaft nicht stabilisieren, und Großbritannien war dazu nicht mehr in der Lage (Kindleberger, 1973).

Die Ursachen für die Große Depression und ihre Folgen finden sich in erster Linie in wirtschaftspolitischen Fehlern, die wiederum auf falschen ökonomischen Vorstellungen, spezifischen Interessen und fehlenden Institutionen basierten. Einige Parallelen zur Konstellation in der EWU drängen sich auf.

## Wirtschaftspolitische Handlungsfelder in der EWU

Für eine erfolgreiche Wirtschaftspolitik ist das kooperative Zusammenspiel der drei großen makroökonomischen Spieler, der Geld-, der Fiskal- und der Lohnpolitik, notwendig. In diesem Bereich weist die EWU strukturelle Defizite auf. Die Fiskalpolitik muss mittelfristig expansiv ausgerichtet bleiben. Dies wird allerdings kontrovers und mit offenem Ergebnis diskutiert. Eine zu schnelle Sparpolitik würde allen Erfahrungen nach in eine längerfristige Stagnation, wenn nicht sogar in eine tiefe Krise führen. Jedoch ist es in der EWU darüber hinaus dringend erforderlich, dass das fiskalische Zentrum der EWU gestärkt wird. Angesichts der hohen Staatsverschuldung einiger EWU-Staaten ist der gemeinsame Wiederaufbauplan von 750 Mrd. Euro nicht ausreichend.

Vollständig unterbelichtet ist in der wirtschaftspolitischen Debatte eine Lohnpolitik, die deflationäre Entwicklungen verhindert. Bereits in der Vergangenheit haben sich die Lohnstückkosten in kaum einem Mitgliedstaat funktional entwickelt (Heine und Herr, 2021, 71 ff.). Dieser Befund spiegelt wider, dass, im Unterschied zu allen anderen Währungsräumen der Welt, für die EWU insgesamt keine den Arbeitsmarkt stabilisierenden Institutionen und Instrumente existieren. Es gibt keine EWU-weiten Absprachen zwischen den Tarifparteien, es gibt keinen koordinierten Mindestlohn, die nationalstaatlich ausgerichteten Gewerkschaften sind unterschiedlich durchsetzungsstark und verfolgen divergierende Strategien. Daher ist nicht auszuschließen, dass im Verlauf einer hartnäckigen Krise der „Lohnanker“ reißt. Um dies zu verhindern, wäre zumindest eine koordinierte EWU-Mindestlohnpolitik flankiert durch gemeinsame arbeitsmarktpolitische Maßnahmen wie etwa ein Kurzarbeitergeld oder Programme gegen die extreme Jugendarbeitslosigkeit in einigen Mitgliedsländern erforderlich.

Die EWU ist des Weiteren schlecht gewappnet, eine interne Finanzmarktkrise mit hohen faulen Krediten, die sich in einzelnen Mitgliedsländern konzentrieren, zu bekämpfen. Notwendig wäre die Vollendung der Bankenunion mit einer gemeinsamen Einlagensicherung und einem machtvolleren Abwicklungsgremium, das auf einen weitaus größeren Abwicklungsfonds zurückgreifen kann. Die für Finanzdienstleistungen zuständige EU-Kommissarin Mairead McGuinness forderte Ende 2020 erneut die schon lange auch vom Chef der Europäischen Bankenaufsicht bei der EZB ins Spiel gebrachten „Bad Banks“: Sie sollen, staatlich gestützt, faule Kredite von den Banken übernehmen und damit deren Bilanzen entlasten (The Guardian, 2020). Es ist zu befürchten, dass bei unterschiedlich ausgeprägten Finanzmarktkrisen in den einzelnen EWU-Ländern erneut politische Streitigkeiten eine schnelle Lösung verhindern.

Angesichts der schwachen konjunkturellen Entwicklung betreibt die EZB seit Jahren eine äußerst lockere Geldpolitik. Zugleich finanziert sie als Kollateralschaden Spekulationen auf den Aktien- und Immobilienmärkten. Ihr fehlen die geeigneten Instrumente einer direkten Bekämpfung auch von offensichtlichen Vermögensblasen. Bisher konnte die EZB das Politikversagen der anderen Makrobereiche im Wesentlichen kompensieren, wenn auch auf Kosten steigender Kollateralschäden. Sie wird auch künftig öffentliche Haushalte indirekt günstig finanzieren müssen und das Finanzsystem bei Liquiditäts- und sogar Solvenzproblemen unterstützen, auch auf die Gefahr hin, damit sogenannte Zombie-Banken oder Zombie-Unternehmen zeitweilig am Leben zu erhalten. Anderenfalls würde sie den Bestand der EWU aufs Spiel setzen. Die Grenze einer solchen Geldpolitik wäre dann erreicht, wenn, aus welchen Gründen auch immer, das Vertrauen in den Euro zusammenbrechen und es zu Kapitalflucht, massiver Abwertung und dadurch zu Inflation kommen würde. Da allerdings die USA eine ähnliche Politik verfolgen und es zum Euro kaum eine Alternative gibt, ist ein solches Szenario in absehbarer Zeit höchst unwahrscheinlich. Aus politischen Gründen könnte jedoch die beschriebene Politik der EZB in schwieriges Fahrwasser gelangen.

Schließlich sollten die Verschuldungsquoten strukturell begrenzt werden. Möglichkeiten werden seit langer Zeit folgenlos diskutiert. So könnten eine korrigierte Einkommens- und Vermögensverteilung oder höhere, progressive Steuereinnahmen die Verschuldung bei den Haushalten und dem Staat begrenzen. Eine langfristige Strategie, in der die EZB allein die Last der Krisenbekämpfung zu tragen hätte, wäre unbefriedigend und gefährlich.

**Title:** ***European Monetary Union: Badly Prepared for Big Crises***

**JEL Classification:** *E6, H12, J3*
